# A Case of Immunoglobulin G4-Related Disease with Extensive Multiorgan Involvements

**DOI:** 10.1155/2015/392893

**Published:** 2015-05-26

**Authors:** Kazuhiko Higashioka, Kenji Yoshida, Kensuke Oryoji, Kazuo Kamada, Shinichi Mizuki, Hiroshi Tsukamoto, Eisuke Yokota, Koichi Akashi

**Affiliations:** ^1^Division of Rheumatology, Matsuyama Red Cross Hospital, 1 Bunkyo-tyo, Matsuyama, Ehime 790-0826, Japan; ^2^Department of Medicine and Biosystemic Science, Graduate School of Medical Sciences, Kyushu University, 3-1-1 Maidashi, Higashi-ku, Fukuoka 812-8582, Japan

## Abstract

We report a case of IgG4-related disease (IgG4-RD) with multiple ten-organ involvement. This case showed many clinical findings, such as bilateral swelling of salivary and lacrimal glands, autoimmune pancreatitis, interstitial nephritis, retroperitoneal fibrosis, periaortitis, systemic swelling of lymph nodes, pulmonary lesions, splenomegaly, and jejunal lesions. He was suspected as having SLE or malignant lymphoma but diagnosed as having IgG4-RD by the elevated serum IgG4 level and histological findings from kidney and lymph node. We report a case of IgG4-RD with multiple ten-organ involvement that was successfully treated with prednisolone therapy.

## 1. Introduction

IgG4-related disease (IgG4-RD) is characterized histologically by dense infiltration of IgG4-positive cells and fibrosis. The first report of the disease came from Japan in 2001 [1]. In this report, Hamano et al. found that patients with autoimmune pancreatitis and sclerosing cholangitis have elevated serum concentrations of the IgG4 subclass, and the level of serum IgG4 decreased significantly during glucocorticoid therapy. IgG4-RD has recently been recognized as an autoimmune systemic disorder since the elevated serum IgG4 levels and dense infiltration of IgG4-positive cells were also reported in Mikulicz's disease, interstitial nephritis, inflammatory pseudotumor, and retroperitoneal fibrosis. We report a patient who presented with extensive clinical findings such as bilateral swelling of salivary and lacrimal glands, autoimmune pancreatitis, interstitial nephritis, retroperitoneal fibrosis, periaortitis, enlargement of lymph nodes, pulmonary lesions, splenomegaly, and jejunal lesions. Few cases appear to have shown extensive multiorgan involvements as this case.

## 2. Case Report

A 64-year-old Japanese man complained of swelling of the submandibular glands in November 2013, and his physician decided to observe the symptoms conservatively after computed tomography (CT) revealed no abnormalities other than submandibular glands. However, swelling of submandibular glands did not improve. A blood sample in April 2014 revealed positive results for anti-nuclear antibody and pancytopenia. Connective tissue diseases such as systemic lupus erythematosus (SLE) were suspected and he was referred and admitted to our hospital in April 2014. He had never smoked, reported no allergies, and had drunk two glasses of beer daily for 30 years. He had undergone bilateral cataract surgery at 55 years old. No history of disease other than cataracts was elicited.

**Figure 1:**
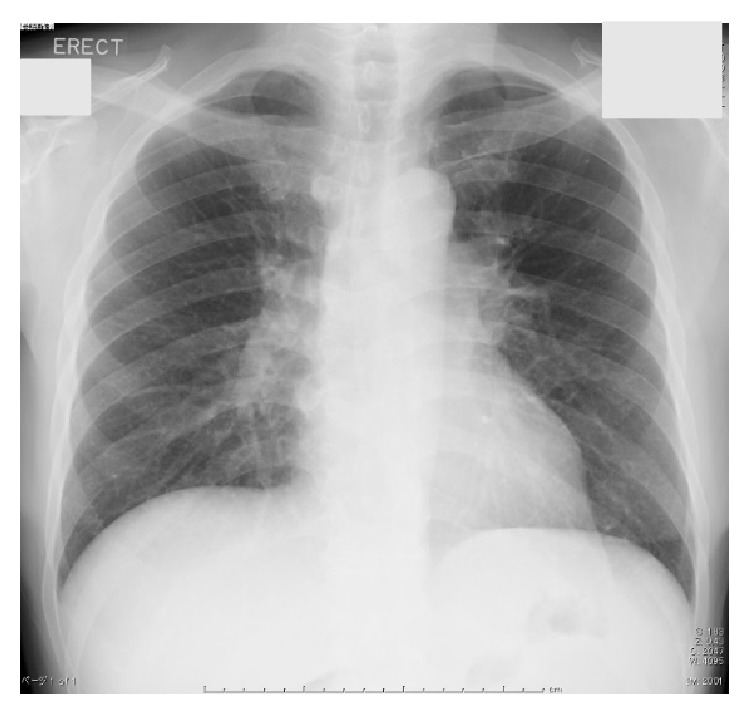
Chest radiography. Reticular and nodular shadows in both lower lobes of the lungs and enlargement of the pulmonary hilum.

**Figure 2:**
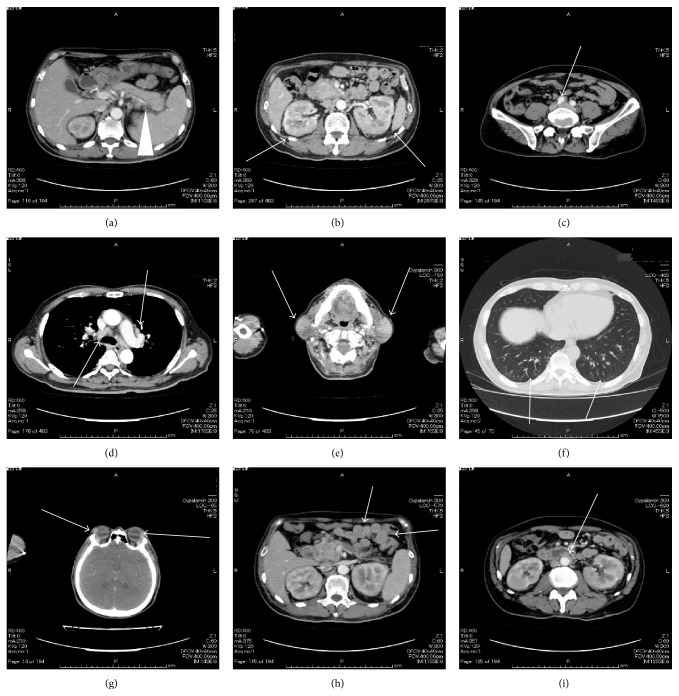
Contrast-enhanced computed tomography. It showed diffuse enlargement of the pancreas which involved something like a capsule that did not show enhancement in the early stage and showed enhancement in the late stage around the rim of the body and caudal portion ((a), white arrow), splenomegaly ((a), white arrowhead), multiple, small, low-attenuation lesions with swelling in bilateral kidneys ((b), white arrows), soft tissue shadow around the right common iliac artery ((c), white arrow), swelling of mediastinal and pulmonary hilar lymph nodes ((d), white arrows), swelling of bilateral submandibular parotid glands ((e), white arrows), centrilobular shadows in both lower lobes ((f), white arrows), swelling of the lacrimal glands ((g), white arrows), edematous changes of jejunum ((h), white arrows), and soft tissue around the abdominal aorta ((i), white arrow).

**Figure 3:**
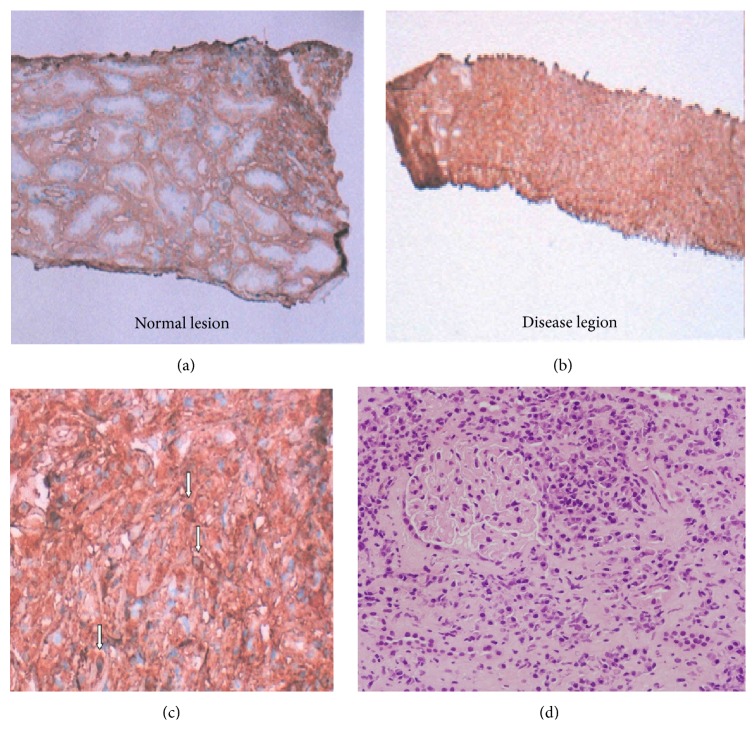
Histopathological findings of the specimen obtained by needle renal biopsy. IgG4 staining (a, b, c) revealed infiltration of numerous IgG4-positive cells and absence of normal structure in some parts of the interstitium and almost-normal structures with only slightly edematous interstitium in other parts. The IgG4/IgG ratio of infiltrating plasma cells was over 90%. Formation of crescents and mesangium cell proliferation were not evident in glomeruli in HE staining (d).

On admission, findings were body temperature, 36.1°C; blood pressure, 109/67 mmHg; heart rate, 89 beats/min; and SpO2, 99% (room air). Physical examination showed symmetrical enlargement of the submandibular and parotid glands, which were nontender, elastic, and soft. Inguinal lymph nodes appeared swollen bilaterally. Laboratory investigations revealed pancytopenia, renal dysfunction, extremely elevated serum levels of IgG and IgG4 subclass, and hypocomplementemia (Table 1). Anti-nuclear antibody was strongly positive and the pattern was homogenous, but anti-histone antibody and Coombs test were not conducted. In addition, IgE was not measured. Chest radiography revealed reticular and nodular shadows in both lower lobes of the lungs and enlargement of the pulmonary hilum (Figure 1). Contrast-enhanced CT revealed various findings in many organs (Figure 2), including bilateral swelling of the submandibular, parotid, and lacrimal glands; enlargement of mediastinal, pulmonary hilar; and inguinal lymph nodes and splenomegaly. Imaging also showed an enlarged pancreas, which involved something like a capsule that did not show enhancement in the early stage but showed enhancement in the late stage around the rim of the body and caudal portion. This image finding was compatible with autoimmune pancreatitis. Both kidneys were enlarged and had many portions of parenchyma that were unenhanced. Soft tissue shadows were seen around the abdominal aorta and the right common iliac artery, suggesting periaortitis and retroperitoneal fibrosis. Centrilobular shadows were seen in both lower lobes in the pulmonary fields and edematous regions were also evident at the jejunum. These findings were thought to be affiliated respiratory and digestive legions. Results of electrocardiography, echocardiography, and brain scintigraphy were normal. Renal histological examination revealed infiltration of numerous IgG4-positive cells and absence of normal structure in some parts of the interstitium and almost-normal structures with only slightly edematous interstitium in other parts (Figure 3). The IgG4/IgG ratio of infiltrating plasma cells was over 90%. Deposits of complement, electron-dense deposits, and immune complex in fluorescent antibody technique were not observed in glomeruli. Formation of crescents and mesangium cell proliferation were also not evident.

**Figure 4:**
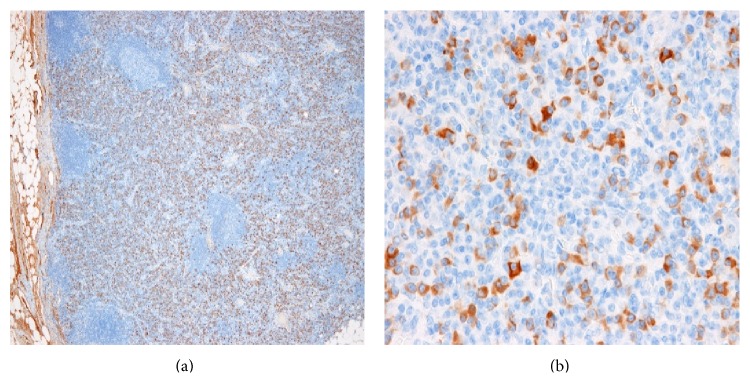
Histopathological findings of the specimen obtained by inguinal lymph node biopsy. IgG4 staining (a, b) revealed IgG4-positive mononuclear cell infiltrates. IgG4/IgG ration was over 90% and more than 300 IgG4-positive plasma cells per high-power field: (a) 4x; (b) 40x.

These clinical, serological, and histological findings helped us to diagnose as IgG4-RD. We carried out bone marrow puncture to rule out multiple myeloma because of extremely high level of serum IgG (M protein was negative.). Plasma cells were slightly increased in bone marrow (3.8%); however, dysplasia or increased blast cells were not seen. Flow cytometry immunophenotyping of bone marrow and peripheral blood was not conducted. Multiple myeloma was able to be ruled out. We also performed biopsy of an inguinal lymph node to rule out malignant lymphoma, because of the elevated serum level of soluble IL-2 receptor. Some cells appeared slightly dyskaryotic, but we could not observe for deviation to one side or the other about light chain in the immunostaining and a proliferative quality destroying the normal structure was not seen. Malignant lymphoma was thus able to be ruled out. Numerous IgG4-positive cells were seen and IgG4/IgG ratio was over 90%. The number of IgG4-positive cells was over 300 cells/high-power field (Figure 4). Histological findings of lymph nodes were compatible with IgG4-RD.

**Figure 5:**
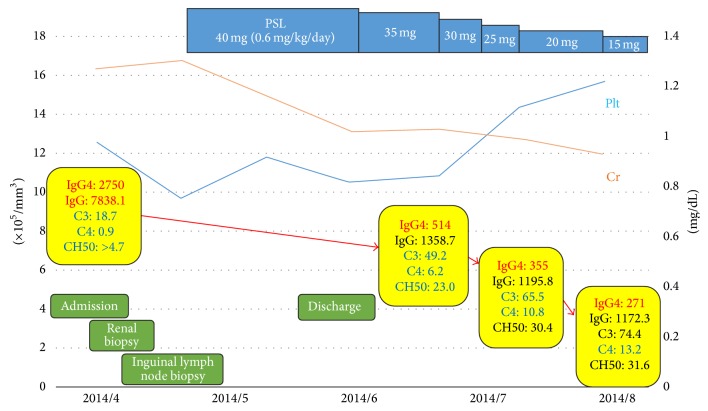
Clinical course of the patient. After admission in April 2014, renal and inguinal lymph node biopsies were performed. We diagnosed IgG4-RD by clinical findings, high level of IgG4, and histological findings in kidney and inguinal lymph node; then prednisolone (0.6 mg/kg/day) was started as therapy. Swelling of the salivary glands improved rapidly and pancytopenia and renal disorder likewise showed improvement. The high levels of serum IgG and IgG4 and hypocomplementemia all also showed marked improvement. These improvements continued until August 2014. The prednisolone dose could be gradually taped.

**Figure 6:**
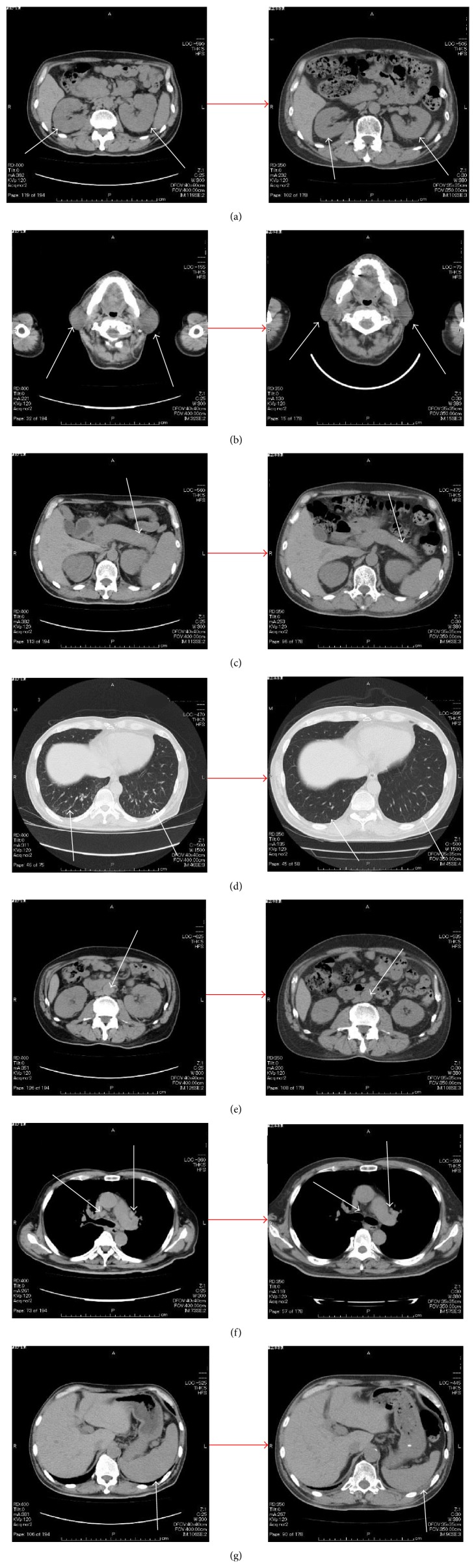
Computed tomography. It showed the improvement of renal enlargement ((a), white arrows), swelling of bilateral salivary glands ((b), white arrows), enlargement of the pancreas ((c), white arrows), centrilobular shadows ((d), white arrows), soft tissue shadows around the aorta ((e), white arrows), mediastinal and pulmonary lymph nodes ((f), white arrows), and splenomegaly ((g), white arrows).

Oral prednisolone (0.6 mg/kg/day) was started as treatment for IgG-RD (Figure 5). Swelling of the submandibular and parotid glands improved rapidly and pancytopenia and renal disorder likewise showed improvement. The patient was discharged at the beginning of June and the prednisolone dose was gradually tapered in outpatient care. The high levels of serum IgG and IgG4 and hypocomplementemia all showed marked improvement 2 months later. Occult blood and protein in urine were also improved. We conducted CT 3 months later for evaluation. We could not perform enhanced CT due to rash after the previous enhanced CT. Imaging showed improvements of the salivary glands, lymph nodes, lacrimal glands, autoimmune pancreatitis, interstitial nephritis, centrilobular shadows in both lower lobes in lungs, periaortitis, and retroperitoneal fibrosis (Figure 6). Edematous changes were no longer apparent in the jejunum and splenomegaly was improved. At the 4-month follow-up, his illness had not recurred. The dose of prednisolone was 10 mg/day in November 2014.

## 3. Discussion

IgG4-RD has been gradually recognized for the last decade. It is characterized histologically by dense infiltration of IgG4-positive cells and fibrosis, but whether infiltration of IgG4-positive cells is the reason for IgG4-RD or a response to some other inflammatory stimulations remains unclear. Few data exist on the global incidence and prevalence of IgG4-RD [2]. Many studies pertaining to the epidemiology of the disease have come from Japan. The prevalence of this disease in Japan has been estimated to be 0.82 per 100,000 persons, but this is likely to be an underestimate given the low level of clinical recognition of this disorder [3, 4].

IgG4-RD has been described in virtually every organ system, including the salivary glands, lacrimal glands, lungs, thyroid, lymph nodes, central nervous system, pituitary body, aorta, pancreas, liver, gall bladder, bile ducts, retroperitoneum, kidney, and prostate [2]. The present case involved as many as ten organs, most of which developed within 5 months (between November 2013 and April 2014). Multiorgan disease may be evident at diagnosis, but usually evolves metachronously over months to years [2]. Fujinaga et al. summarized 90 cases of autoimmune pancreatitis [5]. They found extrapancreatic lesions in 92.2% of the case, including lacrimal and salivary gland lesions (47.5%), lung hilar lymphadenopathy (78.3%), pulmonary lesions (51.2%), thickening of the bile duct wall (77.8%), retroperitoneal fibrosis (19.8%), and renal lesions (14.4%). Kawano et al. summarized 41 cases of IgG4-related renal disorders [6]. They found that the mean number of injured organs was 3.4 for cases involving kidney lesions (range, 1–8). They also found lesions of the salivary glands in 70.7%, lymph nodes in 42.5%, pancreas in 31.7%, lacrimal glands and lung in 29.3%, and retroperitoneum in 7.8%. Smaller numbers of cases involved the prostate, aorta, mammary glands, liver, nerves, thyroid, bile duct, and peritoneal and joint lesions. Only a case was described to involve ten organs: periorbital tissue, salivary glands, lymph nodes, pancreas, kidneys, bile duct, wall of the aorta, prostate, retroperitoneum, and gall bladder [7]. Conversely, some cases have appeared to involve only a single organ disorder as IgG4-RD. Some cases have been reported to involve organs not typically associated with IgG4-RD, such as the heart (a lesion caused complete atrioventricular block [8]), skin [9], superior vena cava [10], hard palate [11], small intestine [12], esophagus [13], and colon [14].

In this case, he was suspected as having SLE because of hypocomplementemia and strongly positive result for anti-nuclear antibody, but 23.4% cases of IgG4-related disease also showed positive result for anti-nuclear antibody and about one-third of IgG4-related disease also showed hypocomplementemia [15]. The male-to-female ratio is said to be 1 : 10 in SLE and a peak age of SLE onset is to be from 20's to 40's. This case did not show anti-ds-DNA antibody and fulfilled all the conditions (clinical, serological, and histological findings) of IgG4-related disease. IgG4-related disease was thought to be a main clinical condition in this case.

The differentiation of IgG4-RD as a systemic disease from multicentric Castleman's disease or granulomatosis with polyangiitis is important. Hyper-IgG4 gammaglobulinemia and IgG4 plasma cell infiltration with fibrosis and sclerosis of tissues may be present in these diseases; thus discriminating between these diseases and IgG4-RD will be difficult in some cases. An IgG4/IgG cell ratio of >50% in the tissue, good response to glucocorticoids, and histological findings of obliterative phlebitis and a storiform pattern suggest IgG4-RD [16, 17].

Glucocorticoids are the first line of therapy and most cases show good response, displaying improvement within a few weeks [18]. A consensus statement from referral centers in Japan has suggested treating patients with IgG4-related pancreatitis initially using prednisolone at a dose of 0.6 mg/kg/day for 2–4 weeks [19]. Cases involving multiorgan have also shown good response to glucocorticoid, with all organs injured by the disease revealing improvements and disappearance of the legions [7, 20, 21]. Our case showed likewise good response to glucocorticoid. In the case which is resistant to glucocorticoid therapy, we have to take account of using immunosuppressants with glucocorticoid and reevaluate the diagnosis of IgG4-RD. Selection of immunosuppressants for controlling this disease remains unclear. Agents such as azathioprine, calcineurin-inhibitors, and mycophenolate mofetil are selected, but their efficacy has yet to be confirmed in clinical trials. One report found that rituximab, anti-CD20 monoclonal antibody, was effective and showed prednisolone-sparing effects larger than those of other immunosuppressants [22]. More cases need to be accumulated to clarify these issues.

In this case, we performed inguinal lymph node biopsy to rule out malignant lymphoma. We showed that the patient was not suffering from malignant lymphoma, but careful follow-up will still be needed because the standardized incidence ratio was 16.0 (95% CI 3.3–45.5) [23], suggesting an increased risk of non-Hodgkin lymphoma among patients of IgG4-RD. In some cases, patients with IgG4-RD have developed diffuse large B cell lymphoma [23], marginal zone lymphoma, follicular lymphoma [24], and anaplastic large B cell lymphoma [25] during follow-up.

In conclusion, we encountered a case with IgG4-RD that had extensive multiorgan involvements with good response to glucocorticoid. Few cases with extensive multiorgan involvements as in this case have been reported. We always have to keep in mind that this disease can happen in any organs.

## Figures and Tables

**Table 1:** Laboratory data.

〈Complete blood count〉	
WBC (*μ*L)	3200
Seg (%)	43.0
Lym (%)	38.0
Mono (%)	18.0
Eos (%)	1.0
RBC (×10^4^/*μ*L)	393
Hb (g/dL)	11.9
Ht (%)	34.7
Plt (×10^4^/*μ*L)	12.5
〈Urine〉	
Sugar	(−)
Protein	(±)
(g/day)	0.56
OB	(2+)
WBC	(−)
〈Chemistry〉	
TP (g/dL)	11.1
Albumin (%)	23.0
*α*1 (%)	2.4
*α*2 (%)	4.5
*β* (%)	3.0
*γ* (%)	67.1
M protein	(−)
T.Bil (mg/dL)	0.3
AST (U/L)	19
ALT (U/L)	9
LDH (U/L)	146
ALP (U/L)	194
*γ*-GTP (U/L)	14
ChE (U/L)	162
KL-6 (U/mL)	117
CK (U/L)	12
BUN (mg/dL)	21.6
Cr (mg/dL)	1.27
UA (mg/dL)	7.1
AMY (mEq/L)	103
Na (mEq/L)	133
K (mEq/L)	4.5
Cl (mEq/L)	102
Ca (mg/dL)	8.4
P (mg/dL)	3.9
〈Coagulation〉	
PT-INR (INR)	1.10
APTT (sec)	27.2
Fib (mg/dL)	317.0
D-dimer (*μ*g/mL)	0.86
〈Immunology〉	
CRP (mg/dL)	0.65
ESR (1 h) (mm)	74
Anti-CCP Ab (IU/mL)	1.8>
RF (mg/dL)	1>
C3 (mg/dL)	18.7
C4 (mg/dL)	0.9
CH50 (U/mL)	4.7>
IgG (mg/dL)	7838.1
IgG4 (mg/dL)	2750
IgA (mg/dL)	108.6
IgM (mg/dL)	193.3
sIL-2R (U/mL)	3562
HBsAg	(−)
HBcAb (S/CO)	0.16
HCVAb	(−)
Anti-dsDNA Ab	(−)
Anti-U1-RNP Ab	(−)
Anti-Sm Ab	(−)
Anti-nuclear Ab	1280 ×
Homogeneous	1280 ×
Speckled	40> ×
Nucleolar	40> ×
Centromere	40> ×
Peripheral	40> ×
Plasma type	(−)
*β*2-GPI Ab (U/mL)	1.3>
LAC	1.0
Anticardiolipin IgG (U/mL)	7

CRP: C-reactive protein; ESR: erythrocyte sedimentation rate; sIL-2R: soluble interleukin-2 receptor; *β*2-GPI Ab: *β*2-glycoprotein I antibody; LAC: lupus anticoagulant.
